# Comparison between Wavefront-optimized and corneal Wavefront-guided Transepithelial photorefractive keratectomy in moderate to high astigmatism

**DOI:** 10.1186/s12886-018-0827-x

**Published:** 2018-06-26

**Authors:** Ikhyun Jun, David Sung Yong Kang, Samuel Arba-Mosquera, Jin Young Choi, Hyung Keun Lee, Eung Kweon Kim, Kyoung Yul Seo, Tae-im Kim

**Affiliations:** 1 The Institute of Vision Research, Department of Ophthalmology, Yonsei University College of Medicine, 50-1 Yonsei-ro, Seodaemungu, Seoul, 03722 South Korea; 2 Corneal Dystrophy Research Institute, Department of Ophthalmology, Yonsei University College of Medicine, Seoul, South Korea; 3 Eyereum Eye Clinic, Seoul, South Korea; 4 Biomedical Engineering Office, Research and Development, SCHWIND Eye-Tech-Solutions, Kleinostheim, Germany

**Keywords:** Transepithelial photorefractive keratectomy, Wavefront-optimized, Corneal Wavefront-guided, Astigmatism

## Abstract

**Abstract:**

**Background:**

To compare the clinical outcomes of wavefront-optimized (WFO) transepithelial photorefractive keratectomy (trans-PRK) and corneal wavefront-guided (CWFG) trans-PRK for myopic eyes with moderate to high astigmatism.

**Methods:**

One hundred ninety-six eyes (196 patients) with moderate to high astigmatism (≥ 1.75 D) treated with WFO or CWFG trans-PRK (101 and 95 eyes, respectively) were retrospectively registered. Safety, efficacy, predictability, vector analysis, and corneal aberrations were compared between groups preoperatively and at 6 months postoperatively.

**Results:**

At postoperative 6 months, the mean logMAR uncorrected distance visual acuity was similar in the WFO (− 0.07 ± 0.08) and CWFG (− 0.07 ± 0.07) groups. Safety, efficacy, and predictability of refractive and visual outcomes were also similar. The correction indices were 1.02 ± 0.14 and 1.03 ± 0.13 in the WFO and CWFG groups, respectively, with no significant difference. The absolute values of the angle of error were significantly higher in the WFO group (2.28 ± 2.44 vs. 1.40 ± 1.40; *P* = 0.002). Corneal total root mean square higher-order aberrations and corneal spherical aberrations increased postoperatively in both groups; however, the change was smaller in the CWFG group. Corneal coma showed a significant increase postoperatively only in the WFO group.

**Conclusions:**

WFO and CWFG trans-PRK are safe and effective for correcting moderate to high astigmatism. However, CWFG trans-PRK provides a more predictable astigmatism correction axis and fewer induced corneal aberrations.

## Background

Transepithelial photorefractive keratectomy (trans-PRK) is an alternative to conventional PRK, and removes the epithelium via laser phototherapeutic keratectomy [1]. Initially, trans-PRK was a two-step surgery in which the corneal epithelium was removed first and then the stroma was ablated; hence, it was not widely used due to longer operating times, higher pain scores, and a deficiency of adequate nomograms [2]. However, single-step trans-PRK (SCHWIND Eye-Tech-Solutions GmbH and Co KG, Kleinostheim, Germany) has been widely used in the field of refractive surgery since its release [3, 4]. This technique combines epithelial ablation, which ablates 55 μm at the center and 65 μm at the periphery, according to a previous epithelial profile study [5], with ablation of the stroma, into a single continuous profile [4]. Several previous studies have shown that transepithelial ablation shortens the operative time and reduces early postoperative pain, haze formation, and the epithelial healing period [3, 4].

Higher-order aberrations (HOAs) have become an important issue in refractive surgery field, because HOAs can affect postoperative visual quality [6]. Glare, halos, haze, or starbursts can occur due to the generation of incidental HOAs after ablation of the cornea. Two treatment profiles, wavefront-optimized (WFO) and wavefront-guided (WFG), are widely employed to reduce the postoperative induction of HOAs [7, 8]. Numerous studies have compared the clinical outcomes in WFO and WFG treatments [6, 7, 9]. Both profiles reduced HOAs and glare symptoms, and increased mesopic contrast sensitivity and patient preference [6, 10–13]. Recently, a study that compared WFO and corneal WFG (CWFG) trans-PRK showed that both modalities are excellent and safe for correction of myopia, while CWFG trans-PRK has some advantages in postoperative HOAs over the WFO profile [1].

However, few studies have compared the astigmatic vector parameters between WFO and WFG treatments [14–16]. Recently, a study comparing the efficacy of astigmatic correction between WFO and WFG profiles in LASIK surgery reported that WFG LASIK resulted in better astigmatism correction and visual outcome compared to that for WFO LASIK [16]. However, reports comparing the clinical outcomes and vector parameters in WFO and WFG for patients with moderate to high astigmatism have not yet been published.

Trans-PRK is an efficient and safe treatment modality for the correction of high myopia [17, 18]; however, there are currently no studies on trans-PRK in patients with high astigmatism. Furthermore, while WFO trans-PRK vector analyses have been reported [17–19], a vector analysis of CWFG trans-PRK has not yet been performed. Thus, the aim of the current study was to evaluate the clinical outcomes, including visual acuity, refractive error, vector parameters, and aberrometric changes, in aberration-free (AF; WFO treatment) and CWFG (WFG treatment) trans-PRK in patients with moderate to high astigmatism.

## Methods

### Subjects

This study is a retrospective, comparative, observational case series, and was approved by the Institutional Review Board of the Yonsei University College of Medicine (Seoul, South Korea; IRB No. 4–2017-0260). The study followed the tenets of the Declaration of Helsinki. The enrolled patients underwent WFO trans-PRK or CWFG trans-PRK performed by a single, experienced surgeon (DSK) at the Eyereum Eye Clinic in Seoul, Korea between October 2014 and February 2017.

The inclusion criteria were as follows: < 10.00 diopters (D) of myopia with ≥1.75 D of refractive astigmatism, 18–40 years of age, stable refraction for at least 1 year, more than 300 μm of residual stromal thickness, and a corrected distance visual acuity (CDVA) of 0.8 Snellen fractions or better. The level of moderate to high astigmatism was set at 1.75 D or more, in accordance with a previous study [20, 21]. All subjects did not wear contact lenses for at least 3 weeks before the preoperative examination. Exclusion criteria were as follows: severe ocular surface disease, including Stevens-Johnson syndrome, graft-versus-host disease, or Sjogren syndrome; corneal epithelial pathology, such as recurrent corneal erosion or epithelial defects; keratoconus; cataracts; previous intraocular or corneal surgery; a history of glaucoma; and any posterior segment pathology. We retrospectively reviewed the medical records of 196 eyes (196 patients) satisfying the study criteria. The right or left eye was randomly chosen, regardless of ocular dominance, refraction, and aberrations.

### Preoperative and postoperative assessments

Before surgery, all patients underwent a detailed ophthalmological examination, including an evaluation of the uncorrected distance visual acuity (UDVA) and CDVA, manifest refraction, slit-lamp examination (Haag-Streit, Köniz, Switzerland), intraocular pressure measurement (noncontact tonometer; NT-530, NCT Nidek Co., Ltd., Aichi, Japan), autokeratometry (ARK-530A autokeratometry; Nidek Co., Ltd., Aichi, Japan), central corneal thickness (CCT) using ultrasound pachymetry (UP-1000;Nidek), and Scheimpflug-based corneal topography (Pentacam HR, Oculus, Wetzlar, Germany). Visual acuity was measured monocularly at 6 m with a Snellen chart (converted to the logMAR scale for statistical analysis). Manifest refraction was performed by one ophthalmologist. Multiple corneal indices were measured at the 8-mm zone using the Scheimpflug tomography system (Pentacam HR; OCULUS). Corneal wavefront aberrations were measured using the Keratron Scout (Optikon 2000, Rome, Italy). The examinations performed preoperatively were also performed at postoperative 1 month, 3 months, and 6 months. The baseline (preoperative) and 6-month postoperative data were analyzed.

### Surgical technique

Ablation profile planning was performed using the integrated Optimized Refractive Keratectomy-Custom Ablation Manager software package (version 5.1, Schwind eye-tech-solutions GmbH and Co KG) and was based on clinical parameters, including manifest refraction, pachymetry, and corneal wavefront data (up to the seventh order; obtained using Keratron Scout). The surgeon could change the optical zone diameter and select the aberrations to be treated. For corneal wavefront–guided treatments, all HOAs were treated with the corneal wavefront–guided ablation profile. A static cyclotorsion compensation algorithm profile was used, and dynamic cyclotorsion control was implemented automatically for all treatments. Centration on the corneal vertex was ensured by input from the topographer. In CWFG treatments, all HOAs were treated using the CWFG ablation profile.

Topical proparacaine hydrochloride 0.5% (Alcaine; Alcon Laboratories, Inc., Fort Worth, TX, USA) drops were instilled in the upper and lower fornices. The eyes were then scrubbed and draped, and a lid speculum was inserted between the lids of the eye to be treated. The other eye was occluded. The epithelium and stroma were ablated using a single continuous profile with the SCHWIND Amaris 1050RS excimer laser platform. Ablation profiles used were the aspheric AF (WFO treatment) or customized full corneal WFG ablation profiles, calculated using the ORK-CAM software module (SCHWIND eye-tech-solutions GmbH and Co KG).

Postoperatively, Mitomycin-C (0.02%) was applied for 30 s, and the eye was then irrigated. Topical levofloxacin 0.5% (Cravit; Santen Pharmaceutical, Osaka, Japan) was applied at the surgical site, and a bandage contact lens (Acuvue Oasys; Johnson & Johnson Vision Care, Inc., Jacksonville, FL, USA) was fitted on to the cornea for 4–5 days until epithelial healing was complete. After surgery, topical levofloxacin 0.5% and fluorometholone 0.1% (Flumetholon; Santen Pharmaceutical, Osaka, Japan) were applied 4 times per day for 1 month. The dosage was gradually reduced over a period of 3 months.

### Statistical analysis

Results are expressed as mean ± standard deviation or mean ± standard error. Group differences were evaluated using Student’s t-test or the Mann-Whitney U test, according to Levene’s test. Linear regression analyses were performed to compare the achieved versus attempted outcomes. Statistical analyses were performed using SPSS statistics software (version 23; IBM Corporation, Armonk, NY, USA). Differences were considered statistically significant when the *p* value was 0.05 or less.

## Results

The WFO group included 101 eyes, while the CWFG group included 95 eyes. The baseline characteristics are provided in Table 1. There were no significant group differences in laterality, age, or sex. Additionally, the mean preoperative spherical equivalent and cylinder were comparable, and there were no significant group differences in the preoperative UDVA, CDVA, CCT, optical zone, or Pentacam indices. The total ablation zone and the maximum ablation depth were significantly smaller in the WFO group compared to that in the CWFG group, despite the comparable optical zones.

**Table 1:** Characteristics of eyes that underwent WFO trans-PRK and those that underwent CWFG trans-PRK

Characteristics	WFO Trans-PRK	CWFG Trans-PRK	*P* value
Number of eyes	101 (R: L = 50: 51)	95 (R: L = 46: 49)	*.879*
Sex	M: F = 53: 48	M: F = 49: 46	*.900*
Age, years old	22.96 ± 2.82 (20 to 33)	23.99 ± 4.78 (18 to 38)	.070
Refractive errors (D)			
Sphere	−4.91 ± 1.77 (− 8.25 to 0.12)	−4.54 ± 2.16 (− 8.12 to 0.25)	.191
Cylindrical	−2.41 ± 0.64 (− 4.25 to − 1.75)	−2.36 ± 0.62 (− 4.50 to − 1.75)	.593
SE	−6.11 ± 1.78 (− 9.19 to − 1.68)	−5.72 ± 2.08 (− 9.00 to − 1.50)	.155
logMAR CDVA	− 0.06 ± 0.08 (− 0.18 to 0.10)	−0.06 ± 0.07 (− 0.18 to 0.10)	.817
logMAR UDVA	1.30 ± 0.34 (0.40 to 2.00)	1.25 ± 0.33 (0.30 to 1.70)	.234
CCT	541.83 ± 31.02 (480 to 613)	538.79 ± 25.46 (470 to 590)	.455
Optical zone (mm)	6.40 ± 0.25 (6.00 to 6.90)	6.42 ± 0.27 (6.00 to 7.00)	.549
Total ablation zone (mm)	8.07 ± 0.15 (7.57 to 8.4)	8.14 ± 0.20 (7.53 to 8.64)	.005^a^
Maximum ablation depth (μm)	142.01 ± 32.71 (60.02 to 202.00)	153.31 ± 29.61 (65.04 to 200.71)	.012^a^
Q value	−0.33 ± 0.12 (− 0.72 to − 0.10)	−0.32 ± 0.13 (− 0.62 to 0.00)	.844
Index of surface variance	25.59 ± 4.78 (17 to 36)	25.59 ± 4.98 (17 to 38)	.999
Index of height asymmetry	5.99 ± 5.77 (0.1 to 27.8)	4.97 ± 3.06 (0.5 to 14.8)	.312
Index of vertical asymmetry	0.13 ± 0.06 (0.05 to 0.32)	0.13 ± 0.05 (0.04 to 0.27)	.823
Index of height decentration	0.009 ± 0.006 (0.000 to 0.029)	0.008 ± 0.004 (0.001 to 0.020)	.755

Results are expressed as means ± standard deviation

*WFO* wavefront-optimized, *CWFG* corneal wavefront-guided, *Trans-PRK* transepithelial photorefractive keratectomy, *D* diopters, *SE* spherical equivalent, *logMAR* logarithm of the minimum angle of resolution, *CDVA* corrected distance visual acuity, *UDVA* uncorrected distance visual acuity, *CCT* central corneal thickness, ^a^significantly different between aberration-free and corneal wavefront-guided groups using *t*-test

### Visual acuity, efficacy, and safety

**Fig. 1:**
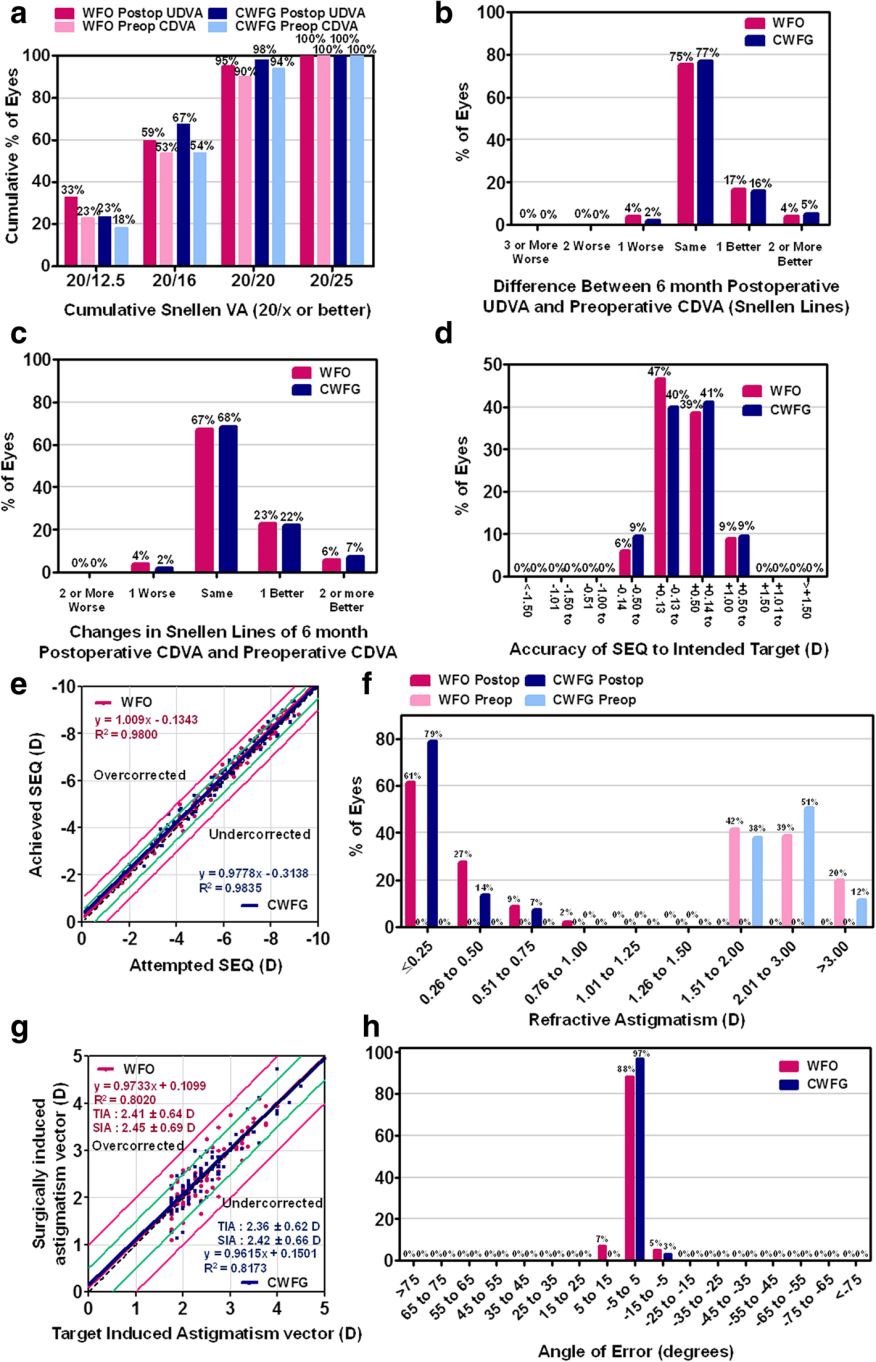
Visual outcomes after WFO and CWFG trans-PRK in moderate to high astigmatism. (**a**) Cumulative 6-months postoperative uncorrected distance visual acuity (UDVA) and preoperative corrected distance visual acuity (CDVA). Snellen line changes in postoperative UDVA (**b**) and CDVA (**c**), relative to preoperative CDVA values, are shown. The accuracy of the spherical equivalent refraction (SEQ) to the intended target (**d**), and attempted versus achieved change in SEQ (**e**) at postoperative 6 months are shown. The relative distribution of preoperative and postoperative 6-months cylinder (**f**) and target-induced versus surgically induced astigmatism (**g**) at postoperative 6 months are shown. (h) The refractive astigmatism angle of error distribution at postoperative 6 months. Data are presented as mean ± standard deviation. WFO, wavefront-optimized; CWFG, corneal wavefront-guided; trans-PRK, transepithelial photorefractive keratectomy

At postoperative 6 months, there were significant improvements in the mean UDVA, relative to preoperative values, in both the WFO (from 1.30 ± 0.34 to − 0.07 ± 0.08) and CWFG groups (from 1.25 ± 0.33 to − 0.07 ± 0.07) (both *p* < 0.001; Tables 1 and 2). In addition, the UDVA was 20/12.5 or better in 33 (33%) and 22 (23%) eyes, 20/16 or better in 60 (59%) and 64 (67%) eyes, and 20/20 or better in 96 (95%) and 93 (98%) eyes in the WFO and CWFG groups, respectively (Fig. 1a). Treated eyes improved in UDVA, in both the WFO and CWFG groups, with 21 (21%) and 20 (21%) eyes gaining one or more lines in the UDVA at postoperative 6 months relative to the preoperative CDVA, (Fig. 1b). None of the eyes in either group lost two or more lines of CDVA at 6 months postoperatively (Fig. 1c). Six months postoperatively, the mean efficacy index (ratio of the postoperative UDVA to preoperative CDVA) was 1.05 ± 0.12 for the WFO group and 1.05 ± 0.12 for the CWFG group, with no significant group difference (*p* = 0.858). The mean safety index (ratio of the postoperative to preoperative CDVA) was 1.07 ± 0.14 for the WFO group and 1.09 ± 0.17 for the CWFG group, with no significant group difference (*p* = 0.586).

**Table 2:** Postoperative visual acuity and refractive errors

	WFO Trans-PRK	CWFG Trans-PRK	P value
logMAR UDVA	−0.07 ± 0.08 (− 0.18 to 0.10)	−0.07 ± 0.07 (− 0.18 to 0.05)	.954
logMAR CDVA	−0.08 ± 0.08 (− 0.18 to 0.10)	−0.09 ± 0.07 (− 0.18 to 0.05)	.896
Sphere (D)	0.35 ± 0.27 (− 0.12 to 1.00)	0.32 ± 0.28 (− 0.25 to 1.00)	.455
Cylindrical (D)	−0.33 ± 0.24 (− 1.00 to 0.00)	−0.27 ± 0.19 (− 0.75 to 0.00)	.076
SE (D)	0.19 ± 0.26 (− 0.38 to 0.88)	0.19 ± 0.27 (− 0.38 to 0.88)	.953

Comparison of postoperative visual acuity and refractive errors between patients who underwent WFO Trans-PRK and those who underwent CWFG Trans-PRK

Results are expressed as means ± standard deviation (range)

*WFO* wavefront-optimized, *CWFG* corneal wavefront-guided, *Trans-PRK* transepithelial photorefractive keratectomy, *logMAR* logarithm of the minimum angle of resolution, *UDVA* uncorrected distance visual acuity, *SE* spherical equivalent

### Refraction

The mean manifest refraction spherical equivalent (MRSE) significantly improved after trans-PRK in both groups (Tables 1 and 2, *p* < 0.001). The accuracy of the refractive correction was excellent (Fig. 1d−g). The achieved SE was within 1.0 D of the intended SE for all treated eyes (both groups). In the linear regression analysis of the attempted versus achieved SE for each technique, the slope and coefficient (R^2^) of were 1.009 and 0.9800, respectively, in the WFO group, and 0.9778 and 0.9835, respectively, in the CWFG group. In terms of astigmatism, 89 (88%) eyes in the WFO group and 88 (93%) eyes in the CWFG group had less than 0.5 D cylinder postoperatively. In the linear regression analysis of the target-induced astigmatism vector versus the surgically-induced astigmatism vector for each technique, the slope and coefficient (R^2^) were 0.9733 and 0.8020, respectively, in the WFO group, and 0.9615 and 0.8173, respectively, in the CWFG group.

### Vector analysis

**Fig. 2:**
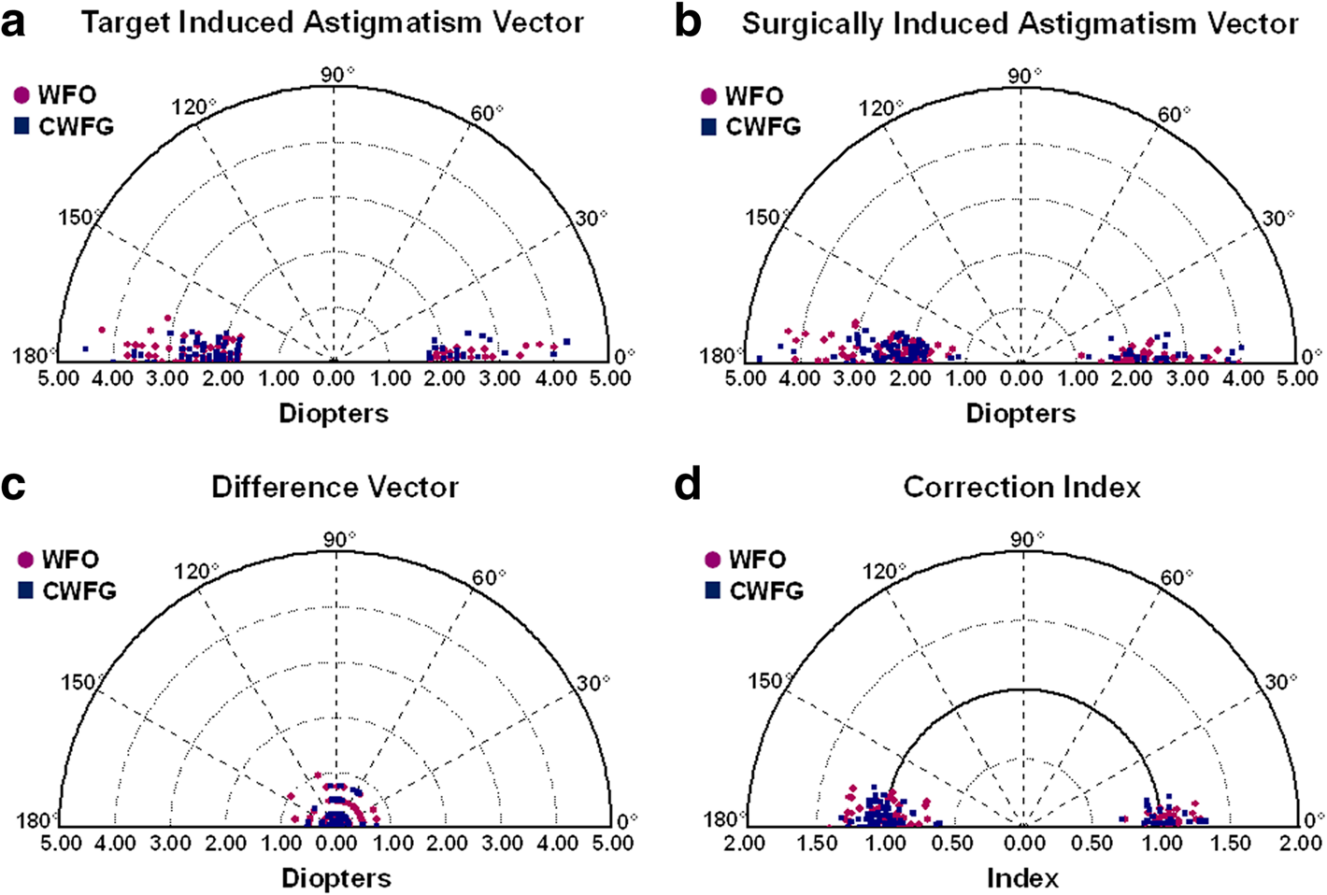
Single angle polar plots of the target-induced astigmatism vector (**a**), surgically-induced astigmatism vector (**b**), difference vector (**c**), and correction index (**d**) after wavefront-optimized (WFO) and corneal wavefront-guided (CWFG) transepithelial photorefractive keratectomy (trans-PRK) at 6 months postoperatively

Using the Alpins method, a vector analysis of astigmatism was performed [22, 23]. Table 3 provides the vector analysis results for the 6-month postoperative refractive data. There were no significant group differences in the target-induced astigmatism (TIA), surgically-induced astigmatism (SIA), difference vector (DV), and correction index (CI) (Table 3). In addition, there were no significant group differences in the index of success (IOS), arithmetic values of the angle of error (AofE), and magnitude of error (MofE). However, the absolute values of the AofE were 2.28 ± 2.44 in the WFO group, and 1.40 ± 1.40 in the CWFG group, with a significant group difference *(p* = 0.002). Because of the symmetric mirror effect in the axis of astigmatism between the right and left eyes [24], we analyzed each eye separately. In the WFO group, the arithmetic values of the AofE were negative for the right eye (i.e., counter-clockwise rotation of achieved correction vectors relative to the original TIAs), but were positive for the left eye (i.e., clockwise rotation of achieved correction vectors relative to the original TIAs). In contrast, the arithmetic values of the AofE were negative for both the right and left eye in the CWFG group. Figure 2 provides polar diagrams of the TIA, SIA, DV, and CI (using the same axes), which were comparable in the two groups.

**Table 3:** Comparison of vector parameters between patients who underwent WFO trans-PRK and CWFG trans-PRK

	Total eye			Right eye			Left eye		
	WFO	CWFG	P value	WFO	CWFG	P value	WFO	CWFG	P value
TIA	2.41 ± 0.64 (1.75 to 4.25)	2.36 ± 0.62 (1.75 to 4.50)	.593	2.43 ± 0.63 (1.75 to 4.00)	2.37 ± 0.66 (1.75 to 4.25)	.632	2.38 ± 0.65 (1.75 to 4.25)	2.35 ± 0.59 (1.75 to 4.50)	.791
SIA	2.45 ± 0.69 (1.11 to 4.25)	2.42 ± 0.66 (1.14 to 4.73)	.722	2.46 ± 0.69 (1.11 to 4.09)	2.44 ± 0.71 (1.14 to 4.73)	.893	2.44 ± 0.70 (1.33 to 4.25)	2.39 ± 0.60 (1.26 to 4.36)	.714
DV	0.33 ± 0.24 (0.00 to 1.00)	0.27 ± 0.19 (0.00 to 0.75)	.076	0.34 ± 0.23 (0.00 to 0.75)	0.30 ± 0.19 (0.00 to 0.75)	.329	0.32 ± 0.26 (0.00 to 1.00)	0.25 ± 0.19 (0.00 to 0.75)	.137
CI	1.02 ± 0.14 (0.63 to 1.41)	1.03 ± 0.13 (0.61 to 1.33)	.742	1.01 ± 0.13 (0.63 to 1.30)	1.04 ± 0.09 (0.61 to 1.33)	.458	1.03 ± 0.15 (0.70 to 1.41)	1.02 ± 0.11 (0.63 to 1.27)	.775
IOS	0.14 ± 0.11 (0.00 to 0.50)	0.12 ± 0.09 (0.00 to 0.40)	.114	0.14 ± 0.10 (0.00 to 0.43)	0.13 ± 0.09 (0.00 to 0.40)	.582	0.14 ± 0.12 (0.00 to 0.50)	0.11 ± 0.08 (0.00 to 0.38)	.106
AofE	− 0.44 ± 3.32 (− 8 to 14)	−0.43 ± 1.93 (− 7 to 5)	.992	−1.36 ± 3.19 (− 8 to 8)	−0.43 ± 1.99 (− 6 to 5)	.089	0.47 ± 3.21 (− 8 to 14)	−0.43 ± 1.90 (− 7 to 3)	.093
|AofE|	2.28 ± 2.44 (0 to 14)	1.40 ± 1.40 (0 to 7)	.002^a^	2.56 ± 2.32 (0 to 8)	1.48 ± 1.38 (0 to 6)	.006^a^	2.00 ± 2.54 (0 to 14)	1.33 ± 1.42 (0 to 7)	.104
MofE	0.05 ± 0.31 (− 0.72 to 0.75)	0.06 ± 0.28 (− 0.74 to 0.74)	.747	0.03 ± 0.29 (− 0.72 to 0.75)	0.08 ± 0.31 (− 0.73 to 0.74)	.475	0.06 ± 0.33 (− 0.71 to 0.74)	0.04 ± 0.26 (− 0.74 to 0.50)	.798

Results are expressed as means ± standard deviation (range)

*WFO* wavefront-optimized, *CWFG* corneal wavefront-guided, *Trans-PRK* transepithelial photorefractive keratectomy, *TIA* target-induced astigmatism, *SIA* surgically induced astigmatism, *DV* difference vector, *CI* correction index, *IOS* index of success, *AofE* angle of error, *|AofE|* absolute value of AofE, *MofE* magnitude of error; ^a^ significantly different between aberration-free and corneal wavefront-guided groups using a student’s *t*-test

### Correlation analyses between refractive correction and preoperative refractive error or offset

**Fig. 3:**
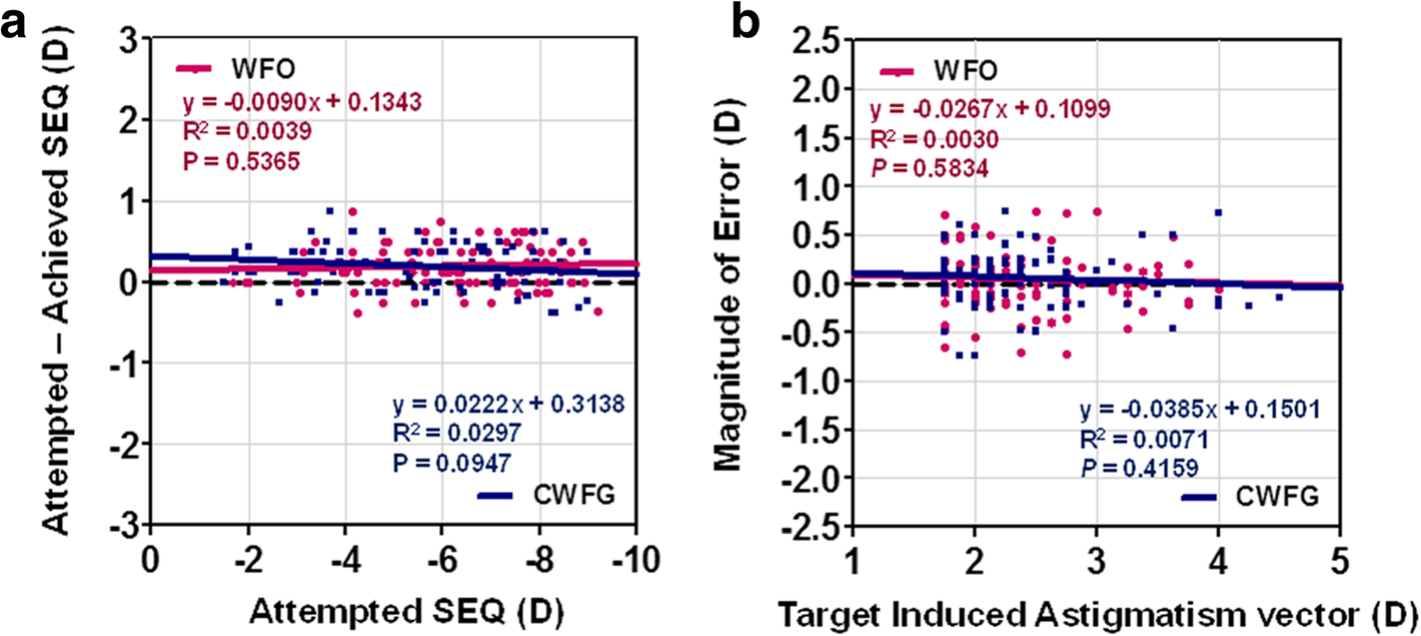
(**a**) SEQ error versus attempted SEQ at 6 months after surgery. (**b**) Magnitude of error versus target-induced astigmatism vector at 6 months after surgery. WFO, wavefront-optimized; CWFG, corneal wavefront-guided; SEQ, spherical equivalent; D, diopter

**Fig. 4:**
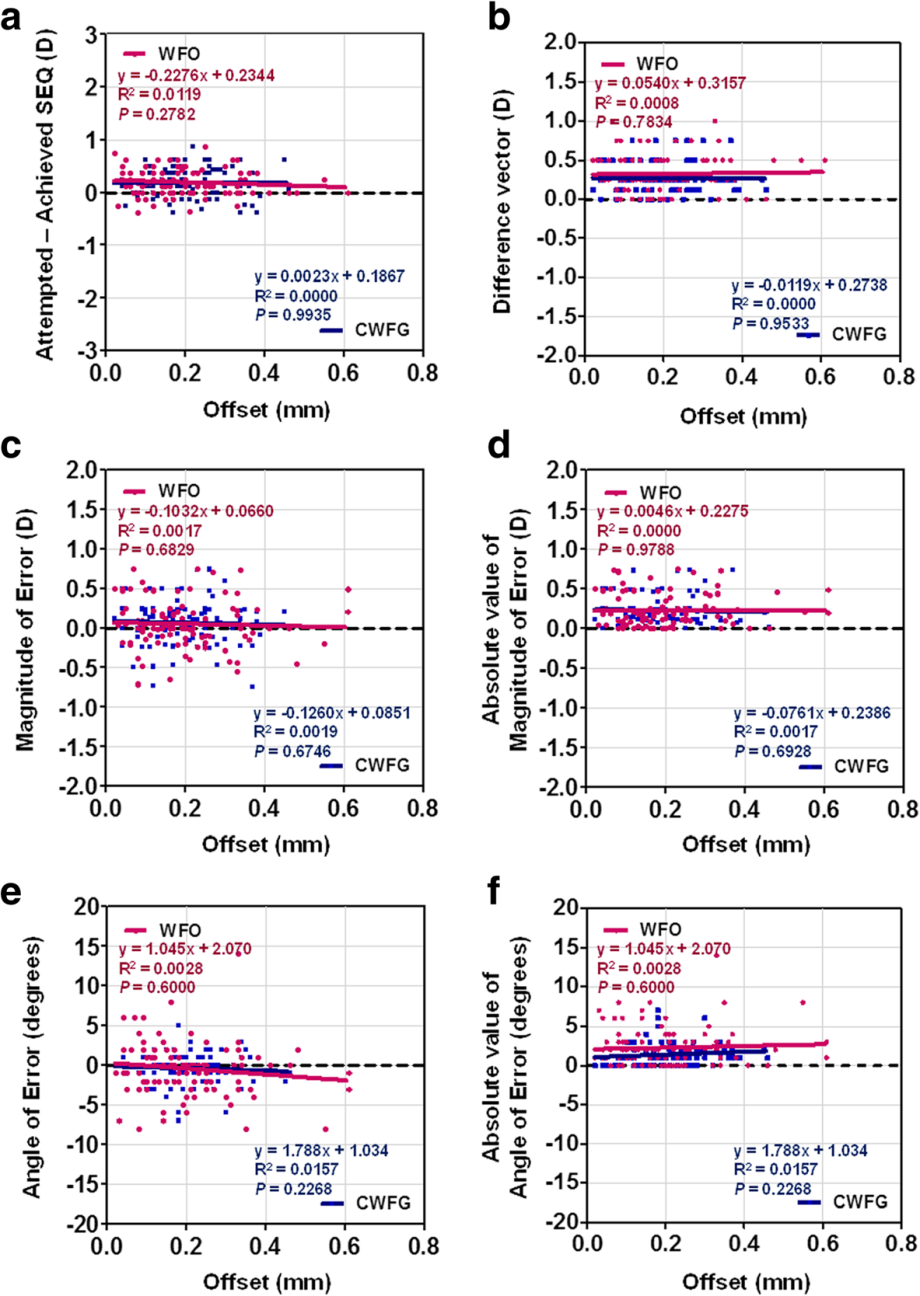
(**a**) SEQ error versus preoperative offset. (**b**) Difference vector versus preoperative offset. (**c**) Magnitude of error versus preoperative offset. (**d**) Absolute value of magnitude of error versus preoperative offset. (**e**) Angle of error versus preoperative offset. (**f**) Absolute value of angle of error versus preoperative offset. WFO, wavefront-optimized; CWFG, corneal wavefront-guided; SEQ, spherical equivalent; D, diopter

There were no significant correlations between attempted SE and SE error (attempted minus achieved SE) in either group (Fig. 3a). Moreover, no significant correlation was noted between TIA and MofE in either group (Fig. 3b). There was no significant interaction between angle kappa and refractive outcomes, including vector parameters (Fig. 4), and no differences were noticed in the linear regression analysis between the WFO group and the CWFG group.

### Higher-order aberrations

**Fig. 5:**
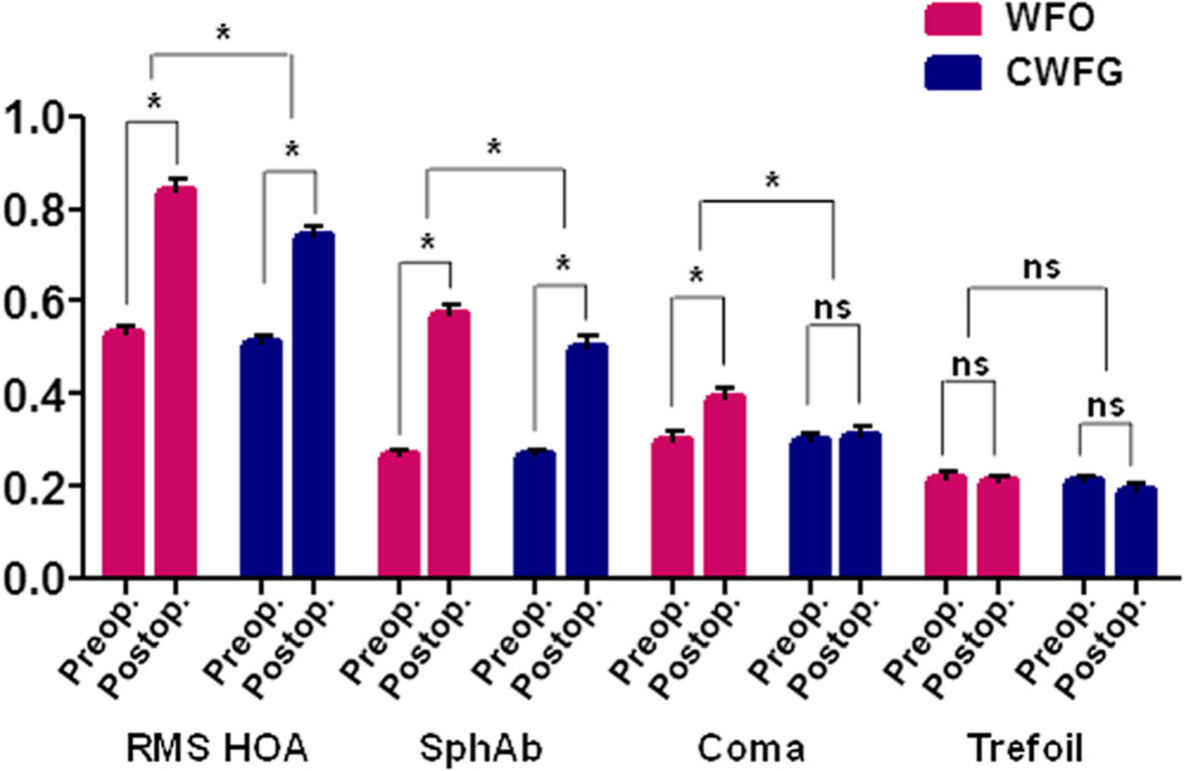
Changes in HOAs at 6 months after WFO and CWFG trans-PRK in moderate to high astigmatism. Data are presented as mean ± SEM. RMS, root mean square; SphAb, spherical aberration; ns, not significant; *, significant. HOAs, higher order aberrations; WFO, wavefront-optimized; CWFG, corneal wavefront-guided; trans-PRK, transepithelial photorefractive keratectomy

Table 4 presents the corneal aberration data. The total corneal RMS HOAs were significantly increased after surgery in both groups (*p* < 0.001). However, the RMS HOAs at 6 months postoperative were significantly smaller in the CWFG group compared to those in the WFO group. Delta values, which represent the change between preoperative and postoperative values, were also significantly smaller in the CWFG group compared to those in the WFO group (Table 4 and Fig. 5). Corneal spherical aberrations significantly increased from preoperative values in both groups (*p* < 0.001). Both the absolute and delta spherical aberration values were significantly smaller in the CWFG group compared to those in the WFO group. Corneal coma increased significantly after surgery in the WFO group (relative to preoperative values), but remained the same in the CWFG group (*p* < 0.001 and *p* = 0.777, respectively). The delta coma values were significantly higher in the WFO group compared to the CWFG group (*p* = 0.007). In contrast, the postoperative corneal trefoil values remained at preoperative levels in both the WFO and CWFG groups, with no significant between-group difference.

**Table 4:** Comparison of corneal aberrations between patients who underwent WFO trans-PRK and CWFG trans-PRK

	RMS HOA			Spherical aberration			Coma			Trefoil		
	WFO	CWFG	*P* value	WFO	CWFG	*P* value	WFO	CWFG	P value	WFO	CWFG	*P* value
Preoperative	0.53 ± 0.15 (0.27 to 1.07)	0.51 ± 0.16 (0.05 to 0.95)	.431	0.27 ± 0.10 (0.10 to 0.51)	0.27 ± 0.11 (0.02 to 0.58)	.989	0.30 ± 0.18 (0.01 to 0.92)	0.30 ± 0.17 (0.02 to 0.77)	.877	0.22 ± 0.11 (0.01 to 0.50)	0.21 ± 0.11 (0.03 to 0.57)	.464
6 month	0.84 ± 0.24 (0.40 to 1.46)	0.74 ± 0.23 (0.27 to 1.71)	.007*	0.57 ± 0.24 (0.04 to 1.10)	0.50 ± 0.26 (0.03 to 1.07)	.040*	0.39 ± 0.21 (0.03 to 0.95)	0.31 ± 0.18 (0.02 to 1.07)	.004*	0.21 ± 0.11 (0.02 to 0.53)	0.19 ± 0.14 (0.01 to 0.67)	.259
P value (vs. preop.)	<.001*	<.001*		<.001*.	<.001*		<.001*.	.777		.526.	.351	
Δ (Pre vs. 6 month)	0.31 ± 0.25 (− 0.18 to 1.04)	0.23 ± 0.26 (− 0.38 to 0.97)	.040*	0.30 ± 0.24 (− 0.23 to 0.86)	0.23 ± 0.24 (− 0.22 to 0.71)	.035*	0.08 ± 0.20 (− 0.41 to 0.63)	0.01 ± 0.20 (− 0.46 to 0.52)	.007*	−0.01 ± 0.12 (− 0.31 to 0.22)	−0.02 ± 0.15 (− 0.43 to 0.32)	.622

Results are expressed as means ± standard deviation (range)

*WFO* wavefront-optimized, *CWFG* corneal wavefront-guided, *Trans-PRK* transepithelial photorefractive keratectomy, *RMS* Root mean square; *HOA* higher-order aberration, *Δ* change; * *P* value < 0.05

## Discussion

WFO and WFG treatments have mainly been evaluated with regard to visual acuity, correction of refractive errors, and HOAs; few studies have compared the treatments using astigmatism vector analysis [14–16]. Furthermore, an evaluation of astigmatic correction in patients with moderate to high astigmatism and a comparison of WFG and WFO profiles in trans-PRK were lacking. Thus, we evaluated the clinical outcomes and vector parameters after WFO and CWFG trans-PRK in myopic eyes with moderate to high astigmatism.

Similar to previous studies [1, 4, 25, 26], the postoperative outcomes of WFO and CWFG trans-PRK in our study demonstrate that both methods are efficient and safe for treating moderate to high astigmatism. The treatments did not significantly differ in postoperative visual acuity or refractive errors. In addition, the efficacy and safety indices were comparable and there was no significant relationship between the attempted SE and SE error in either group (Fig. 3a).

Moreover, the mean CI values—the ratios of the TIA to the SIA—were approximately 1, and the mean MofE values, which represent the difference between the attempted and achieved astigmatism vector, were low in both groups, indicating that both treatment modalities are equally efficacious and predictable in terms of correction of astigmatism. There was no significant relationship between TIA and MofE in either group (Fig. 3b). A previous study using vector analysis showed good astigmatism correction in trans-PRK, comparable to that for alcohol-assisted PRK [17]. However, we found little difference in the astigmatic correction axis between the two profiles of trans-PRK. The AofE values were significantly higher in the WFO group compared to those in the CWFG group; although the difference might seem small, a 4-degree axis difference can theoretically result in a 14% undercorrection in astigmatism [27]. The slightly larger (nonsignificant; *p* = 0.078) DV values in the WFO group may have been related to the higher values of the absolute AofE. A previous study reported absolute AofE values of 1.9 ± 0.67 for WFG LASIK and 9.7 ± 3.70 for WFO LASIK, with a significant group difference; however the groups did not differ in DV, CI, IOS, and MofE [14]. Kalifa and colleagues also reported that the AofE is significantly better in moderate myopic eyes after WFG LASIK; furthermore, WFG LASIK showed significantly better results in DV, CI, and MofE compared to that for WFO LASIK [16]. Another study examining WFG and WFO PRK reported that WFO PRK elicited a higher absolute AofE and comparable DV, CI, IOS, and MofE to that observed in WFG PRK [15]. Given these results, including the present results, we can infer that the WFG profile is superior to the WFO profile in astigmatism correction, especially in terms of the AofE. In addition, the arithmetic AofE values were negative in the right eye and positive in the left eye in the WFO group in the present study, while the AofE values were negative in both the right and left eyes in the CWFG group. These data suggest that while the WFO profile in trans-PRK can be influenced by eye laterality, the CWFG profile is not.

We analyzed whether the angle kappa is associated with refractive outcomes. There were no significant relationships between offset and refractive outcomes, including SE error, DV, MofE, and AofE (Fig. 4). Both treatments were performed under centration to the corneal vertex; hence, the angle kappa was effectively compensated for, and no difference was noted between the two groups.

Although the corneal RMS HOAs significantly increased after treatment in both groups, the amount of change was significantly larger in the WFO group at 6 months postoperative. Reduced induction of RMS HOAs in the CWFG group can result in better visual quality. Similarly, corneal spherical and coma aberrations were also increased after trans-PRK in both profiles, but to a lesser extent in CWFG trans-PRK. Numerous previous studies have shown that the WFG profile has some advantages in HOAs induction after refractive surgery over the WFO profile, consistent with the results of the present study [1, 7, 16, 28, 29]. There is only one previous study that compared WFO and CWFG trans-PRK; no significant group differences in the induction of spherical aberrations were observed [1]. In contrast, the present study showed greater spherical aberration induction in WFO trans-PRK compared to that in CWFG trans-PRK in patients with moderate to high astigmatism. In addition, corneal coma was significantly increased after surgery in the WFO group, but not in the CWFG group, similar to the results of a previous study [1]. These results suggest that the CWFG profile may have some benefits over the WFO profile in treating moderate to high astigmatism. The total ablation zone and the maximum ablation depth were significantly larger in the CWFG group despite of comparable OZ between the groups, as we previously reported [1]. This might be associated with less induction of HOAs.

The present study has several limitations. Mainly, it was not designed as a prospective randomized trial and had a relatively short follow-up period. Despite these limitations, we believe the current study is of value, as it is the first investigation comparing the WFO and CWFG profiles in eyes with moderate to high astigmatism in terms of clinical outcomes and vector parameters. Thus, our study results can aid surgeons who treat myopic eyes with moderate to high astigmatism using trans-PRK.

## Conclusions

Both WFO and CWFG profiles elicited excellent, safe, and predictable visual outcomes and refraction in the treatment of moderate to high astigmatism. However, the CWFG profile showed better results with regard to the astigmatism correction axis. Moreover, the induced corneal HOAs after treatment were significantly less with the CWFG profile compared to that with the WFO profile.

## Abbreviations

AF: Aberration-free
AofE: Angle of error
CCT: Central corneal thickness
CDVA: Corrected distance visual acuity
CI: Correction index
CWFG: Corneal wavefront-guided
DV: Difference vector
HOAs: Higher-order aberrations
IOS: Index of success
logMAR: Logarithm of the minimal angle of resolution
MofE: Magnitude of error
MRSE: Manifest refraction spherical equivalent
RMS: Root mean square
SEQ: Spherical equivalent refraction
SIA: Surgically-induced astigmatism
TIA: Target-induced astigmatism
trans-PRK: Transepithelial photorefractive keratectomy
UDVA: Uncorrected distance visual acuity
WFO: Wavefront-optimized
